# Impacts of Cigarette Smoking Status on Metabolomic and Gut Microbiota Profile in Male Patients With Coronary Artery Disease: A Multi-Omics Study

**DOI:** 10.3389/fcvm.2021.766739

**Published:** 2021-10-28

**Authors:** Xiaomin Hu, Yue Fan, Hanyu Li, Ruilin Zhou, Xinyue Zhao, Yueshen Sun, Shuyang Zhang

**Affiliations:** ^1^Department of Cardiology, State Key Laboratory of Complex Severe and Rare Diseases, Peking Union Medical College Hospital, Chinese Academy of Medical Science & Peking Union Medical College, Beijing, China; ^2^Department of Medical Research Center, State Key Laboratory of Complex Severe and Rare Diseases, Peking Union Medical College Hospital, Chinese Academy of Medical Science & Peking Union Medical College, Beijing, China

**Keywords:** smoking, coronary artery disease, gut microbiota, metabolomics, sphingolipids metabolism

## Abstract

**Background:** Cigarette smoking has been considered a modifiable risk factor for coronary artery disease (CAD). Changes in gut microbiota and microbe-derived metabolites have been shown to influence atherosclerotic pathogenesis. However, the effect of cigarette smoking on the gut microbiome and serum metabolites in CAD remains unclear.

**Method:** We profiled the gut microbiota and serum metabolites of 113 male participants with diagnosed CAD including 46 current smokers, 34 former smokers, and 33 never smokers by 16S ribosomal RNA (rRNA) gene sequencing and untargeted metabolomics study. A follow-up study was conducted. PICRUSt2 was used for metagenomic functional prediction of important bacterial taxa.

**Results:** In the analysis of the microbial composition, the current smokers were characterized with depleted *Bifidobacterium catenulatum, Akkermansia muciniphila*, and enriched *Enterococcus faecium, Haemophilus parainfluenzae* compared with the former and never smokers. In the untargeted serum metabolomic study, we observed and annotated 304 discriminant metabolites, uniquely including ceramides, acyl carnitines, and glycerophospholipids. Pathway analysis revealed a significantly changed sphingolipids metabolism related to cigarette smoking. However, the change of the majority of the discriminant metabolites is possibly reversible after smoking cessation. While performing PICRUSt2 metagenomic prediction, several key enzymes (wbpA, nadM) were identified to possibly explain the cross talk between gut microbiota and metabolomic changes associated with smoking. Moreover, the multi-omics analysis revealed that specific changes in bacterial taxa were associated with disease severity or outcomes by mediating metabolites such as glycerophospholipids.

**Conclusions:** Our results indicated that both the gut microbiota composition and metabolomic profile of current smokers are different from that of never smokers. The present study may provide new insights into understanding the heterogenic influences of cigarette smoking on atherosclerotic pathogenesis by modulating gut microbiota as well as circulating metabolites.

## Introduction

Cigarette smoking is a major modifiable cardiovascular risk factor (1). Epidemiological evidence has shown that current smokers with coronary artery disease (CAD) undergo percutaneous coronary intervention (PCI) at a younger age and have a significantly higher short-term and 3-year mortality compared with non-smokers (2, 3). However, smoking cessation in patients with CAD can substantially lower the risk of recurrent cardiovascular events and all-cause mortality (4).

In the last few decades, there has been a surge of interest in the pathophysiologic role of gut microbiota in atherosclerosis and cardiovascular disease. Possible mechanisms involve symbiont microbiota influencing the host immune system or generating microbial-derived products such as trimethylamine N-oxide (TMAO) and short-chain fatty acids (SCFAs) (5, 6). TMAO can accelerate the progression of atherosclerosis by enhancing the accumulation of cholesterol in macrophages and foam cells in artery walls as well as enhancing platelet hyperreactivity and thrombosis (7, 8). Moreover, elevated plasma levels of TMAO were associated with an increased risk of major adverse cardiovascular events, which is independent of traditional risk factors, even in the low-risk population (9). Based on bracing discoveries in microbiota-host interaction in atherosclerosis, novel therapeutic targets have been proposed for the treatment of cardiometabolic diseases, such as bacterial enzyme inhibitors and dietary substrate analogs (10, 11).

Cigarette smoking may influence host microbiota through various mechanisms including upregulating oxidative stress-associated enzymes in gut immune cells, altering the gut mucin layer, and increasing the intestinal pH (12–14). A prior study showed that gut microbiome compositions of smokers differed significantly from those of never smokers in the healthy population (15), but whether this change can be reversed by smoking cessation remains a matter of controversy (16, 17). Besides, the smoking-induced intestinal microbiota changes under the background of chronic diseases like Crohn's disease and ankylosing spondylitis have been investigated but whether this change contributes to disease progression is not determined (18, 19). Whereas, little is known about the effect of smoking on gut flora and its association with disease progression in patients with CAD. In this study, we sought to address the knowledge gap by evaluating the effects of smoking status on gut microbiota and serum metabolome to explain the role of smoking on CAD pathogenesis from a multi-omics view.

## Materials and Methods

### Study Participants and Sample Collection

We consecutively recruited patients who were hospitalized for coronary angiography at Peking Union Medical College Hospital (PUMCH). Male patients with ≥50% stenosis in at least one main coronary artery were included in this study. All female patients were excluded since the percentage of current smokers was too low (4/50). Participants were excluded if they had infectious diseases, gastrointestinal diseases, malignant tumors, autoimmune disorders, renal dysfunction (severe renal disease or creatinine >3.0 mg/dl), a history of gastrointestinal surgery in the previous year, or antibiotics usage lasting for more than 3 days in the previous 3 months. A total of 113 male patients with CAD were enrolled and further split into the following three groups: (1) current smokers, (2) former smokers, and (3) never smokers. The coronary atherosclerotic burden of each patient was assessed using the Gensini score by two professional cardiologists as described in our previous publication (20).

After admission, in-hospital participants were given stool samplers and provided with detailed instructions on sample collection. Freshly collected stool samples were immediately transported to the laboratory and frozen at −80°C to prevent microbiota structure shift. Fasting peripheral venous blood was collected in the morning of the day after admission, and all clinical, as well as smoking information, were collected. The study was performed in accordance with the principles of the Declaration of Helsinki. All subjects provided written, informed consent for participation in this study.

### Smoking Information

Smoking information was obtained via questions while hospitalization at PUMCH. Participants who were active smokers at the point of admission were classified as current smokers. Participants who had a smoke history but quitted smoking prior to admission (smoking cessation > 2 months before the time of interview) were defined as former smokers. Smoking intensity (cigarettes per day, only for current smokers) and smoking burden (pack-years) were also collected.

### Sample Collection, 16S rRNA Processing, and Sequencing

Bacterial DNA was isolated from fecal samples by utilizing the bead-beating method and then proceeded to PCR amplification and sequencing of the V3–V4 region of the 16S rRNA gene under raw data quality control. A sequencing library of the V3–V4 regions of the 16S rRNA gene was established. The purified products were mixed at an equal ratio for sequencing using an Illumina MiSeq system (Illumina Inc., USA). EasyAmplicon was utilized for the analysis of downstream amplicon information (21). Operational taxonomic units (OTUs) were delineated at a cutoff value of 97% by using USEARCH v.8.0 after dereplication performed by the *-derep_fullength* command of VSEARCH32 (v2.15) (22). Taxonomic classification of OTUs was achieved using the sintax algorithm of USEARCH based on the Ribosomal Database Project (RDP) training set v16.

### Analysis of the Taxonomic Composition and Prediction of Gut Microbiota Phenotype

The OTU feature table was created with *-usearch_global* command and taxonomic annotation was generated by USEARCH *-otutab* command based on the Greengenes database. Alpha diversity analysis was carried out using the vegan package (v2.5-6) in R v4.0.2 (23). Differences in Shannon's index and the ACE index between groups were evaluated using Tukey's honestly significant difference (HSD) test. The weighted UniFrac distance matrix was generated using *usearch -beta_div*. Beta diversity calculations were performed by principal coordinate analysis (PCoA) and the Adonis test was applied to test for significant differences between groups. The R package ggplot2 was used to visualize the results of the diversity analyses. The taxonomic composition of each group was visualized as a stacked bar plot at the phylum and genus level by the ggplot2 package. For OTU comparisons between groups, EdgeR was utilized to identify significantly differential features and the Benjamini-Hochberg method was applied to control the false discovery rate (FDR).

PICRUSt2 was used to predict the metagenomic functional compositions (24). STAMP software (v2.1.3) was utilized for statistical analyses to compare the microbiota structure at different levels (Welch's *t*-test) and predicted pathways (White's non-parametric test). Linear discriminant analysis (LDA) effect size (LEfSe) (http://huttenhower.sph.harvard.edu/galaxy) was used to compare the gut composition structure.

### Untargeted Metabolomics Study

Sample analysis was performed by a Waters ACQUITY ultra-high-performance liquid chromatography (HPLC) system (Milford, MA) coupled with a Waters Q-TOF Micromass system (Manchester, UK). Sample analysis was performed in both positive and negative ionization modes, while both polar ionic and lipid modes were used depending on the properties of metabolites. The detailed procedures for sample preparation, HPLC-mass spectrometry (MS) experiments, and peak-ion intensity matrix preparation were described in our previous publication (20). The matrix was further reduced by removing peaks with missing values in more than 80% of the samples and those with isotope ions from each group to obtain consistent variables. The coefficient of variation (CV) of metabolites in the quality control (QC) samples was set at a threshold of 30% for the assessment of repeatability in the metabolomics datasets. Partial least squares discriminant analysis (PLS-DA) was applied by SIMCA software (v14.1, Umetrics, Sweden) to calculate variable importance in the projection (VIP) values. Significant differential metabolites were selected on the basis of VIP value > 1 and *p* < 0.05. Annotation and classification of metabolites were achieved by online databases, as described in our previous publication (20). MetaboAnalyst (http://www.metaboanalyst.ca) (version 4.0) was used for the identification of metabolic pathways and analysis.

### Follow-Up Study

Post-discharge, a follow-up study was conducted by return visit at PUMCH or by telephone interviews with patients or close family members. The composite endpoint of this study consisted of all-cause mortality and/or stroke and/or reoccurrence of acute coronary syndrome (ACS) and/or readmission for cardiac causes. The identification of composite endpoint events was based on the electronic medical record system of PUMCH or telephone interviews in cases of events outside PUMCH. Binary logistic regression analysis was employed to explore the relationship between smoking status and the outcome of patients with CAD after adjusting for potential confounding factors using IBM SPSS (v26.0, SPSS Inc., Chicago, IL, USA). The results of binary logistic regression were visualized as forest plots using the R package ggplot2 (25).

### Statistical Analysis

A total of 113 study participants were categorized into three groups: current smokers (*N* = 46), former smokers (*N* = 34), and never smokers (*N* = 33). Spearman correlations between important bacterial taxa, serum metabolomic features, and clinical parameters were calculated in IBM SPSS v.26.0 software. Correlations between features were visualized using the pheatmap R package and corrplot R package. A Sankey plot was utilized to present the multi-omics correlation with the R package networkD3.

## Results

### Characteristics of the Study Population

A total of 113 male participants who were diagnosed with CAD at admission were consecutively enrolled at PUMCH and were further divided into the following three groups based on their smoking status: current smokers (*N* = 46), former smokers (*N* = 34), and never smokers (*N* = 33). The characteristics and traditional cardiovascular risk factors for the participants are summarized in Table 1. In terms of the number of stenosed vessels, we observed that the current smokers and former smokers exhibited a higher proportion of two- or three-stenosed vessels than the never smokers. However, the difference in the Gensini score had no significant difference. In general, the difference in disease severity was inconspicuous in participants with different smoking statuses according to the biochemical data at the baseline.

**Table 1:** Characteristics of the study cohort.

	**Current, *N* = 46**	**Former, *N* = 34**	**Never, *N* = 33**	***p*-values**
Age*	58.48 ± 9.96	61.44 ± 9.3	62.52 ± 11.82	0.198
Systolic blood pressure (SBP) (mmHg)*	131.32 ± 20.43	128.97 ± 13.43	129.54 ± 16.36	0.836
BMI*	26.41 ± 3.22	26.05 ± 3.01	26.00 ± 3.15	0.831
**Type of CAD (%)**				0.604
SCAD	11 (23.9)	11 (32.4)	10 (30.3)	
UA	24 (52.2)	12 (35.3)	16 (48.5)	
MI	11 (23.9)	11 (32.4)	7 (21.2)	
Gensini^#^	32 (14.5, 65.5)	45.0 (23.5,65.8)	32.5 (20.0, 46.0)	0.455
**No. of vessels (%)**				0.043
0	4 (8.7)	0 (0)	12 (6.1)	
1	10 (21.7)	5 (14.7)	13 (39.4)	
2	13 (28.3)	6 (17.6)	7 (21.2)	
3	19 (41.3)	23 (67.6)	11 (33.3)	
**History (%)**				
OMI	4 (8.7)	9 (26.5)	4 (12.1)	0.076
DM	13 (28.3)	10 (29.4)	12 (36.4)	0.724
FLD	9 (19.6)	4 (11.8)	7 (21.2)	0.546
HTN	31 (67.4)	21 (61.8)	18 (54.5)	0.51
**Medication (%)**				
HTNdrug	28 (60.9)	21 (61.8)	18 (54.5)	0.802
OAD	10 (21.7)	7 (20.6)	8 (24.2)	0.934
Statin	15 (32.6)	11 (32.4)	13 (39.4)	0.782
**Laboratory data**				
TC (mmol/L)*	3.97 ± 1.01	3.6 ± 0.63	3.99 ± 1.27	0.176
TG (mmol/L)*	1.92 ± 1.75	1.46 ± 0.61	1.4 ± 0.59	0.103
HDL-C (mmol/L)*	0.89 ± 0.19	0.93 ± 0.21	0.97 ± 0.18	0.235
LDL-C (mmol/L)*	2.28 ± 0.75	2.03 ± 0.58	2.37 ± 1.1	0.218
hsCRP (mg/L)^#^	2.02 (1.04, 3.56)	1.78 (0.48, 4.48)	1.86 (0.54, 7.35)	0.712
cTnI (ng/ml)^#^	0.011 (0.000,0.037)	0.012 (0.000,0.075)	0.003 (0.000, 0.065)	0.706

Values are presented as *mean ± SD, ^#^median (IQR).

*One-way ANOVA and Kruskal-Wallis H-test were employed in cases of continuous data. Categorical variables were compared by the χ^2^ test or Fisher's exact test.*

*SCAD, stable coronary artery disease; UA, unstable angina; MI, myocardial infarction; No. of vessels, number of stenosed vessels;l OMI, old myocardial infarction; DM, diabetes; FLD, fatty liver disease; HTN, hypertension; OAD, oral antidiabetic drugs*.

### Relatively Worse Clinical Outcome of Current Smokers Compared With Former and Never Smokers

Among the enrolled 113 male patients, 106 patients were followed up by interview through phone or electronic medical record and seven patients were out of touch, or personal rejection in rare cases. The median follow-up time was 3.95 [interquartile range (IQR): 3.69–4.27] years. In the current smokers (42/46 successfully followed up), composite endpoint events were observed in 14 subjects, including one cardiac death [myocardial infarction (MI)], 11 recrudescent ACS, and 13 readmissions for cardiac issues. In the former-smoker group (33/34 followed up), composite endpoint events were observed in nine patients, including one non-cardiac death, six recrudescent ACS, and six readmissions for cardiac causes. Lastly, in the never-smoking patients with CAD (31/33 successfully followed up), composite endpoint events were observed in three patients, including one cardiac death (MI) as well as one non-cardiac death, one recrudescent ACS, and one readmission for cardiac causes. Binary logistic analyses demonstrated that current active smoking was associated with an increased risk of ACS reoccurrence [odds ratio (OR) = 10.855, 95% CI: 1.236–95.360, *p* = 0.031] and an increased risk of readmission due to cardiac issues (OR = 7.181, 95% CI: 1.411–36.553, *p* = 0.018) after adjusting for confounding factors, including age, history of old myocardial infarction (OMI), diabetes, oral antidiabetic drugs (OAD), hypertension (HTN), drugs for HTN, and fatty liver disease (FLD) (Supplementary Figures S1A,B, Supplementary Table S1). However, in the binary logistic regression, the *p*-value for former vs. current smokers is not significant for both ACS reoccurrence (*p* = 0.114) and readmission for cardiac issues (*p* = 0.074), indicating statistically no difference between the outcome of former and never group in our study cohort.

### Gut Microbiome Composition in Male Patients With CAD Varies With Different Smoking Status

**Figure 1:**
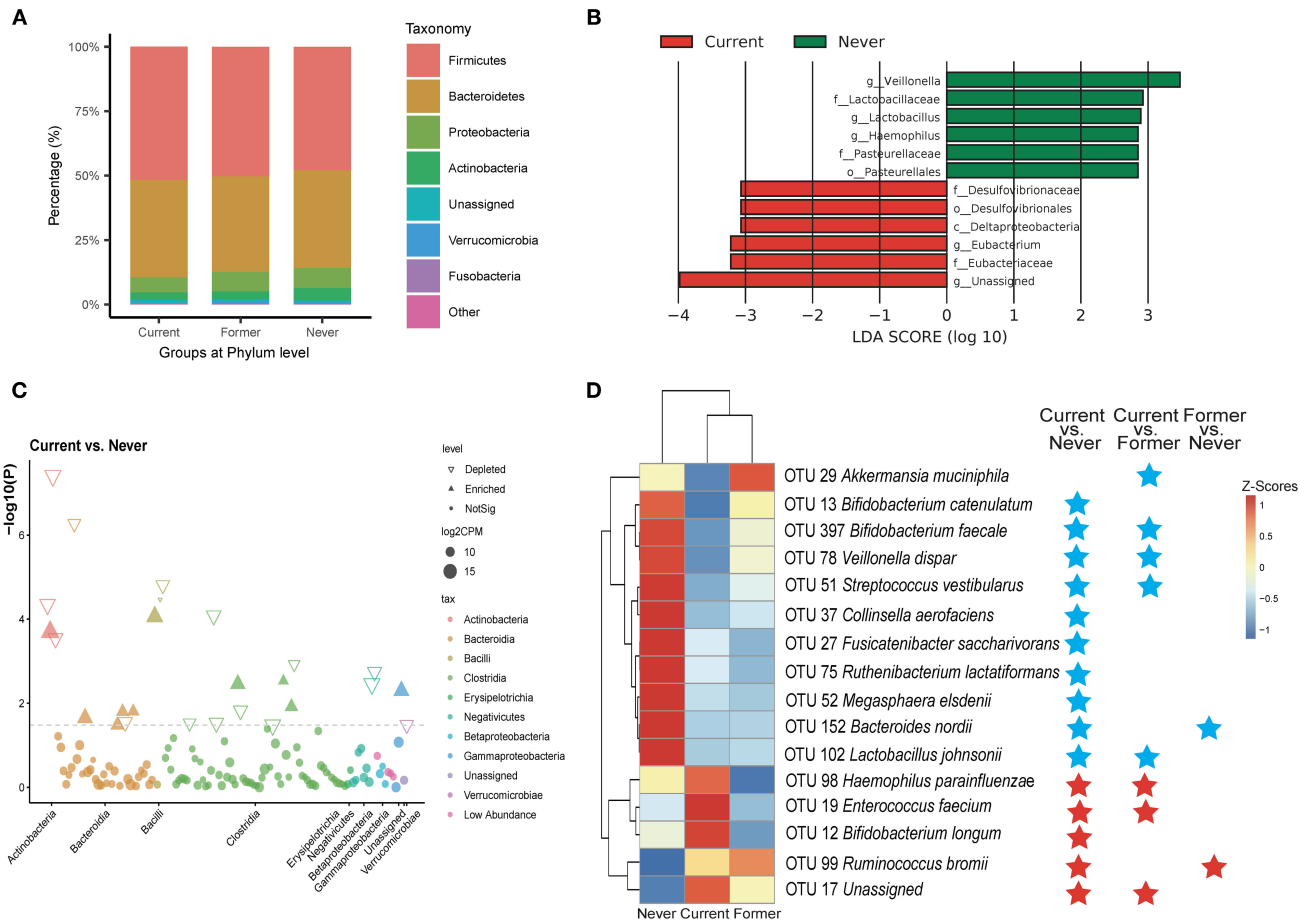
Alterations in the composition of gut microbiota associated with smoking status. **(A)** Dominant phyla in each group. **(B)** Linear discriminant analysis (LDA) effect size (LEfSe) analyses of the differential taxa between current and never smokers. **(C)** A Manhattan plot showing the distribution of differential taxa. **(D)** A heatmap illustrating the relative abundance of smoking-associated operational taxonomic units (OTUs) across the three groups. A threshold of FDR < 0.05 calculated by the edgeR test was considered statistically significant. Blue stars indicate the depleted OTUs in comparison; red stars indicate the enriched OTUs.

In the 16s gut microbiota investigation, a total of 2,830,519 high-quality 16S rRNA reads were obtained, with a median read count of 22,842 (range: 11,202–44,385) per sample. A total of 626 OTUs were obtained by clustering sequences within a percent sequence similarity threshold of 97%. In terms of the diversity of gut microbiota, we observed no significant differences in either alpha or beta diversity (PCoA based on the weighted UniFrac distances) among the three groups (shown in Supplementary Figures S2A,B). At the phylum level, the relative proportions of six phylas were assessed and their contributions in each group are shown in Figure 1A. The percentage of Firmicutes increased and Proteobacteria decreased among current smokers compared with never smokers (relative abundance: Firmicutes 51.7 vs. 46.9%; Proteobacteria 5.9 vs. 7.8%), while the former group was at an intermediate level. It is consistent with the previous report on the increased general abundance of Firmicutes in smokers (16). LEfSe analysis was utilized to compare the bacterial composition between groups. Taxa with LDA scores >2 were displayed in Figure 1B. The bacterial communities were different between current smokers and never smokers, while the latter was characterized by a bloom of members of the *Desulfovibrionaceae*, and decreased *Veillonella* and *Lactobacillaceae*. *Desulfovibrionaceae* was the most discriminant feature for the current smokers (LDA score 3.06, *p* = 0.039), whereas the *Veillonella* genus was the most discriminative for the never smokers (LDA score 3.47, *p* = 0.007). It was reported that *Desulfovibrio* DNA progressively increased with the smoking burden (pack-years) (26). The overall decreased *Veillonella* abundance in smokers at the genus level is in line with an earlier study (15). Manhattan plots showed the contributions of differentially abundant OTUs at the class level (Figure 1C). The comparison of relative abundance at phylum and genus levels is also carried out between current smokers and never smokers, and these discriminant taxa were correlated with clinical indicators (Supplementary Figure S3C, Welch's *t*-test). At the genus level, we found decreased abundance in *Akkermansia* and increased *Roseburia* in current smokers.

To compare the detailed composition differences between groups, edgeR was utilized with a threshold of *p* < 0.05 and FDR < 0.2. A total of 26 OTUs exhibited significantly different abundances in the comparison between the current and never smokers (16 depleted and 10 enriched OTUs), as shown in the volcano plot (Supplementary Figure S2D). These discriminant OTUs with FDR < 0.05 were summarized in Supplementary Table S2 and displayed in Figure 1D. Several smoking-associated OTUs decreased in current smokers belonged to *Bifidobacterium*, such as OTU 397 (*Bifidobacterium faecale*) and OTU 13 (*Bifidobacterium catenulatum*). We noticed that OTU 13 (*B. catenulatum*) is negatively correlated to blood glucose, implying its antidiabetic effect (Supplementary Figure S3A). OTU 29 (*Akkermansia muciniphila*, FDR < 0.001*)* was found to be significantly depleted in current smokers compared with former smokers and is negatively correlated to several inflammation indicators (IL-6&IL-18). OTU 37 (*Collinsella aerofaciens*) showed a negative correlation with low-density lipoprotein cholesterol (LDL-C). The smoking-positive OTUs include potential pathogens such as OTU19 (*Enterococcus faecium*), OTU 98 (*Haemophilus parainfluenzae*), and OTU 32 (*Klebsiella*). OTU 98 (*Desulfovibrio piger)* under the genus *Desulfovibrio* also had an elevated abundance among smokers. We also noticed that OTU 17 (uncultured clone 218002-1-48, belonging to the Lachnospiraceae family) is positively correlated to total cholesterol (TC), triglyceride (TG), LDL-C, and free fatty acid (FFA), indicating the potential detrimental role of OTU 17 in lipid metabolism.

### Untargeted LC-MS Analysis Reveals Smoking-Specific Metabolomic Signatures

We then explored the serum metabolome among different smoker groups by the untargeted LC-MS method. After QC and removal of the low-abundance peaks, metabolomic (polar ionic mode, positive, and negative) and lipidomic (lipid mode, positive and negative) profiling yielded 14,585 (PP, 7,246 annotated), 7,394 (NP, 3,304 annotated), 5,193 (PLP, 1,973 annotated), and 4,974 (LPN, 2,491 annotated) features, respectively. The PLS-DA analyses were carried out to discriminate the metabolomic profiles of current-smoking and never-smoking patients with CAD. The PLS-DA scatter plots under the four modes are shown in Supplementary Figure S4. A total of 304 metabolites (VIP value > 1 and Wilcoxon rank-sum *p*-value < 0.05) whose abundance significantly changed in current smokers compared to never smokers were selected, including 248 features annotated and classified based on the online databases (Supplementary Table S3). The VIP values of the top 20 discriminant metabolites are visualized in a bar plot in Supplementary Figure S5.

**Figure 2:**
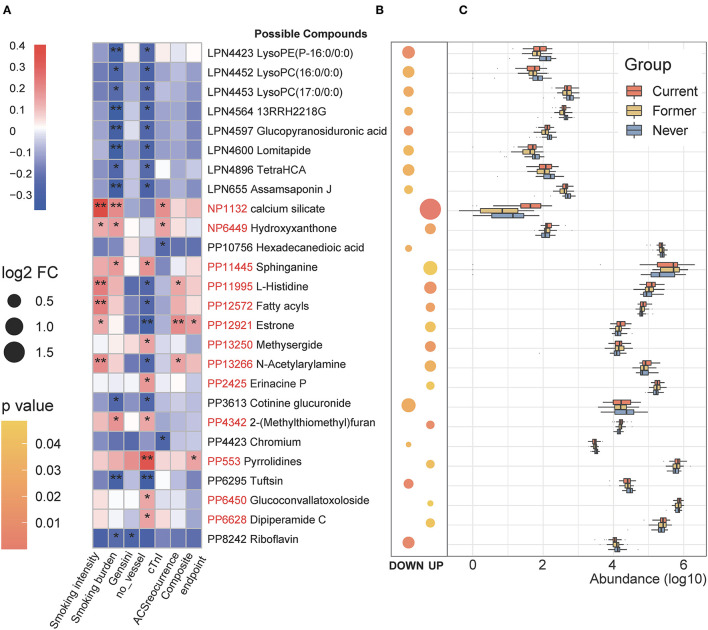
Identification of the major smoking-associated serum metabolomic features correlated with clinical phenotypes. **(A)** Spearman correlations between smoking-associated metabolic features and clinical outcomes as well as indicators of disease severity in patients with CAD. *p < 0.05, **p < 0.01. **(B)** Fold of change and *p*-values of important metabolomic features. The left column shows the smoking-negative metabolomic features; the right column shows the smoking-positive metabolites. Bubble size indicates the relative fold change in comparison between current and never smokers. Log2 FC, Log2 (the fold of change), Current vs. Never smokers. **(C)** Changes in important smoking-associated metabolomic features among the three groups.

We subsequently assessed the correlation between the smoking-related serum metabolites and clinical indicators with special attention to the smoking intensity and burden. As shown in Figures 2A, 24 metabolites were significantly correlated with the indicators of CAD severity [evaluated by Gensini score, number of stenosed vessels, and cardiac troponin I (cTnI) levels] and the follow-up outcomes. And the fold of change and concentration of these differential metabolites are displayed in Figures 2B,C. Notably, PP553 (belonging to pyrrolidines) was identified to be positively related to disease severity as well as clinical outcomes. In contrast, we observed that three smoking-negative (decreased in current smokers) metabolic features (LPN4423, LPN4452, and LPN4453, all belonging to glycerophospholipids) and LPN4896 (TetraHCA, belonging to the class bile acid) were negatively correlated with disease severity and adverse outcomes of patients (*p* < 0.05, Spearman correlation). Compounds including PP12572, PP13266 (N-acetylarylamine), PP12921 (Estrone), and PP11995 (L-Histidine) were shown to be strongly related to smoking intensity and positively related to the adverse clinical outcomes. PP8242 (Riboflavin) is another smoking-related metabolite of interest in our study, which is negatively related to smoking burden and Gensini score. Earlier researches have demonstrated that smoking can induce downregulation in circulating vitamin B, including riboflavin and the deprivation of riboflavin may aggravate cardiovascular illnesses (27, 28).

**Figure 3:**
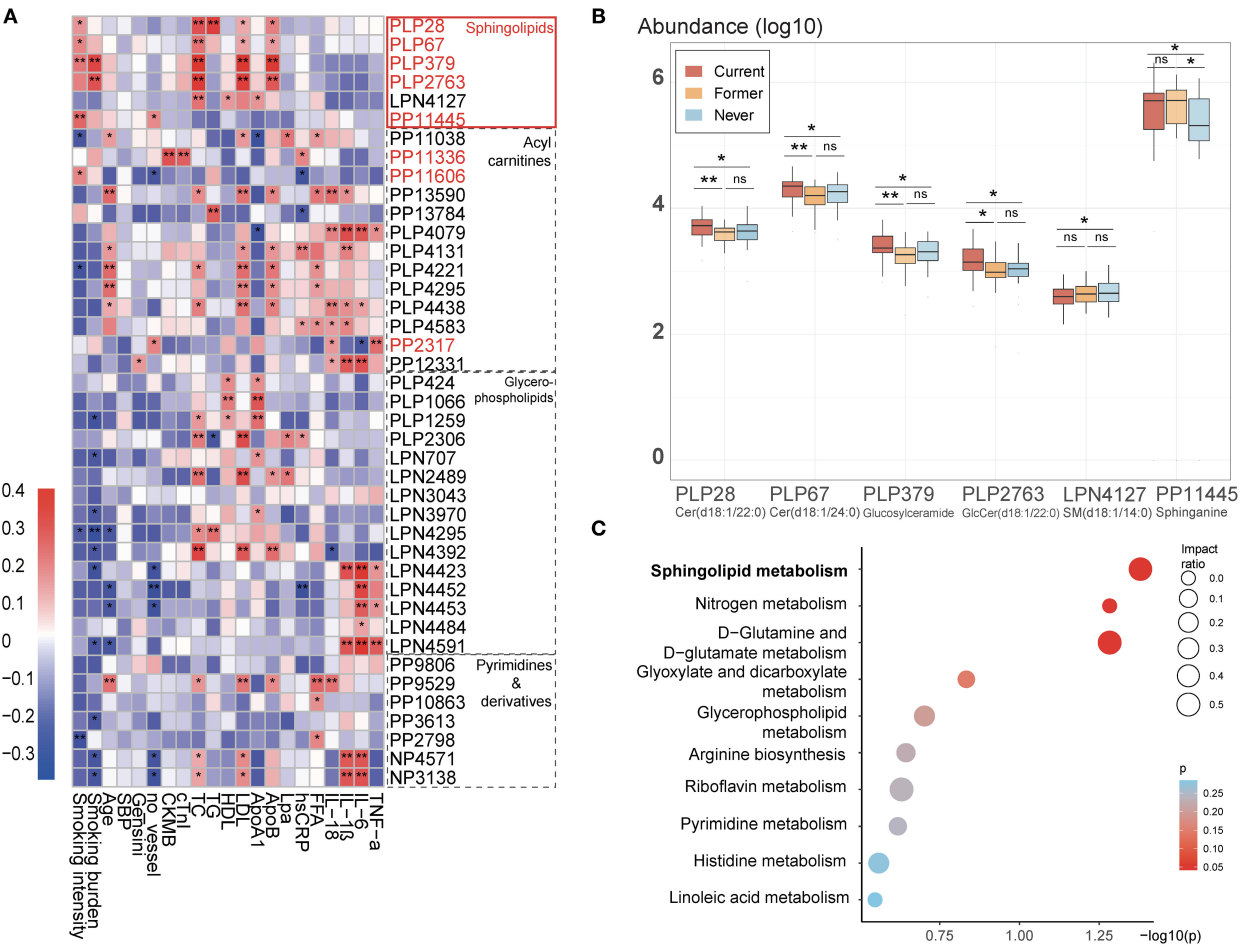
Changes of four groups of important smoking-associated serum metabolomic features. **(A)** Spearman correlations between smoking-associated metabolomic features in four main groups and major risk factors for CAD. The IDs of metabolic features highlighted in red are smoking-positive. Lpa, lipoprotein (a); ApoB, apolipoprotein B; FFA, free fatty acid; ApoA1, apolipoprotein A1. There is a remarkable elevation in sphingolipids, which is correlated with CAD phenotypes. *p < 0.05, **p < 0.01. **(B)** Relative abundance of six metabolites belonging to the class of sphingolipids among three groups. ns: not significant, *p < 0.05, **p < 0.01, Mann-Whitney *U* test. **(C)** Pathway analysis of differential metabolites (Current *vs*. Never smokers).

**Figure 4:**
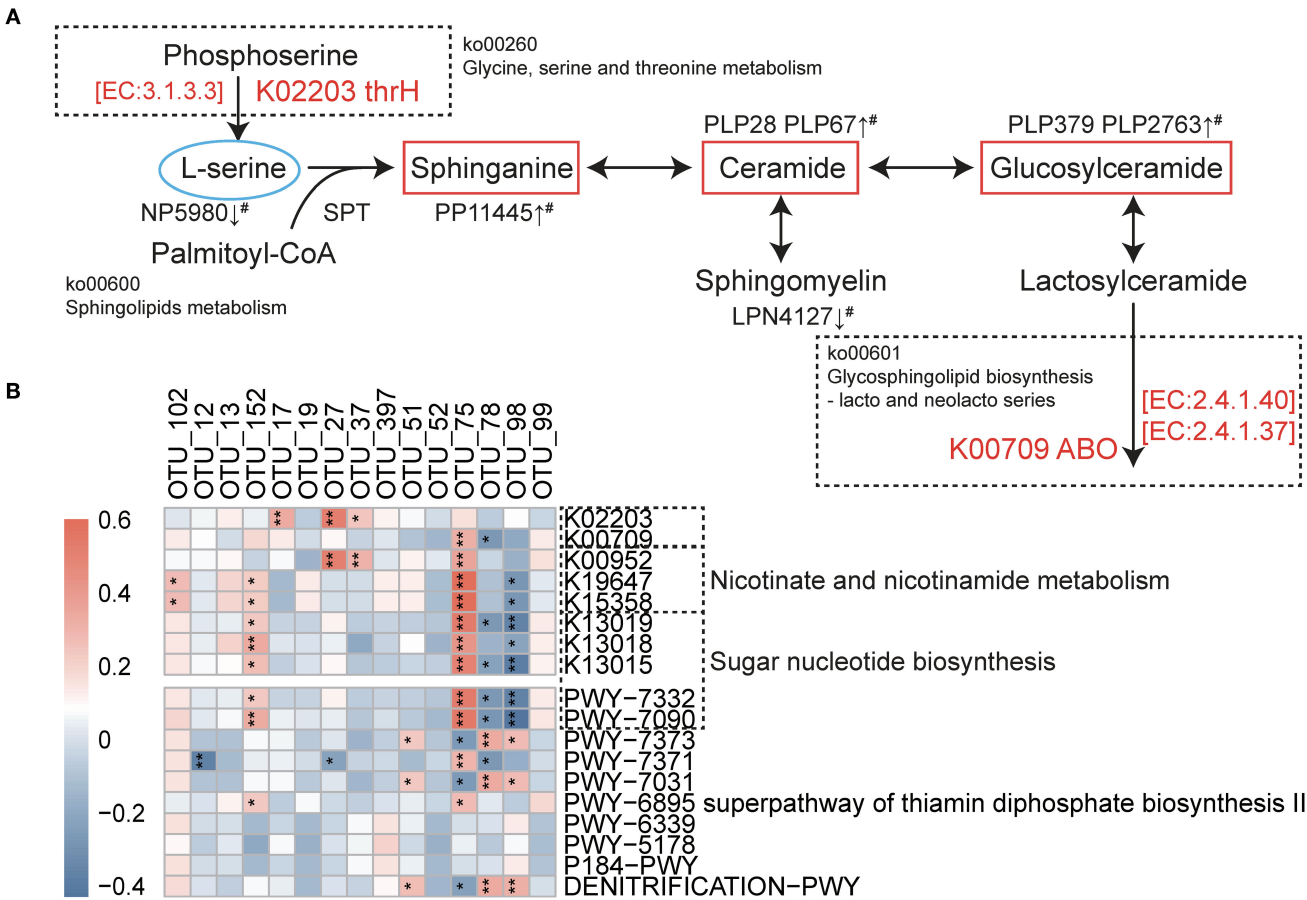
Association between predicted metagenomic functional changes and differential OTUs as well as sphingolipids metabolism. **(A)** Relationship between sphingolipids pathway and two related differential enzymes (K02203 and K00709) predicted by PICRUSt2. The features are highlighted in red (smoking-positive) or blue (smoking-negative). ^#^Observed metabolomic changes in smokers with CAD compared with never smokers in the present study. IDs and names of enzymes and pathways were based on the KEGG database. SPT, serine pamitoyl-transferase; K02203, homoserine phosphotransferase; K00709, histo-blood group ABO system transferase. **(B)** Spearman correlations between smoking-associated OTUs and metagenomic prediction including differential pathways as well as important enzymes. IDs of enzymes were according to the KEGG database; IDs of pathways were according to the MetaCyc database. *p < 0.05, **p < 0.01.

Figure 3A presented the discriminant smoking-related metabolites in four major groups (including sphingolipids, fatty acyl carnitines, glycerophospholipids, and pyrimidine and derivatives) and their relationship with clinical parameters. Notably, the sphingolipid family is strongly correlated to the lipidomic profile among patients with CAD. PLP 28 [Ceramide(d18:1/22:0)], PLP 67 [Ceramide(d18:1/24:0)], PLP 379 (Glucosylceramide), and PLP 2,763 [GlcCer(d18:1/22:0)] are all correlated with smoking burden or intensity and LDL-C, TC, and apolipoprotein B (ApoB). The smoking-negative LPN4127 [SM(d18:1/14:0)] was positively corelated to cardioprotective HDL and ApoA1, implying the general lipidotoxic effect of shifts in sphingolipids induced by smoking. Among the six sphingolipids, PP11445 (Sphinganine) is positively correlated to the number of stenosed vessels but interestingly negatively correlated to the relative abundance of the Phylum Bacteroidetes (Rho = −2.48, *p* = 0.008, Spearman correlation), which will be explained in the discussion part. Furthermore, we found a declined L-serine in current smokers, which also plays a vital role in sphingolipids metabolism. The relative abundances of the identified metabolites involved in sphingolipids metabolism across different groups are shown in Figure 3B, while the mutual conversion between the sphingolipids is summarized in a simplified pathway map in Figure 4A. 9-Decenoylcarnitine, median-chain acyl carnitine, which is positively associated with smoking and tightly correlated with MB isoenzyme of creatine kinase (CKMB), cTnI, and hsCRP in our study, was reported that to be associated with incident atrial fibrillation (29).

We also noticed the depletion of several bile acids (PP7591, LPN4896, PP1057, LPN3069, and PP6142) as well as taurine (NP6888) in smokers. The previous study has shown that microbial enzymes include bile salt hydrolase (BSH) and bile acid-inducible (BAI) enzymes are essential for bile acid homeostasis in the host, which has a further influence on the host lipidomic profile (30). We correlated the genera capable of deconjugation (*Lactobacillus, Bacteroides*, and *Bifidobacterium*) and dehydroxylation (*Clostridium, Eubacterium*) with three groups of differential smoking-related metabolites (bile acids, sphingolipid, and glycerophospholipids) to investigate the possible interrelationship (31). The correlation map was visualized with corrplot R package in Supplementary Figure S6. Generally, the genera capable of bile acid transformation were negatively correlated to the abundance of the metabolites, especially the glycerophospholipids. Among the genera, *Eubacterium* and *Bacteroides* showed a tighter correlation with glycerophospholipids.

### Further Comparison Between Former and Never Smokers Revealed the Reversibility of Metabolomic Changes

*T*-tests were also performed between the former smokers and never smokers to further explore the effect of smoking cessation behavior on the serum metabolomic profile (Supplementary Table S3). Seventy out of 304 metabolites remained differential metabolites (*p* < 0.05, Former vs. Never), while 234 showed no significant differences, revealing the partial reversibility in metabolomic change after smoking cessation. Most of the irreversible metabolites were correlated with smoking burden instead of smoking intensity. Some of these metabolites correlated tightly with the inflammation indicators.

The related metabolic pathway analysis was performed on MetaboAnalyst 4.0. Among the top 10 pathways, sphingolipid metabolism had the most significant *p*-value and a relatively big impact ratio. Other involved pathways include D-Glutamine and D-glutamate metabolism and Glycerophospholipid metabolism (Figure 3C, Supplementary Table S4). The critical irreversible pathways generated with 70 possibly irreversible metabolites mainly comprise glycerophospholipid, sphingolipid, and linoleic acid pathways.

### Prediction of Bacterial Metagenomic Functions Associated With Smoking Status

The functional potentials of the gut bacterial community were predicted using the PICRUSt2 tool based on the MetaCyc database (24), including pathway prediction and enzyme functional prediction. A total of 10 pathways were found to differ in the pairwise comparison among the three groups (Supplementary Figures S7, S8, Supplementary Tables S5, S6), and these smoking-related pathways were correlated with the discriminant OTUs. The smoking-positive pathways are mainly involved in sugar nucleotide biosynthesis (PWY-7332 and PWY-7090), whose downstream pathways include D-Glutamine and D-glutamate metabolism. PWY-7090 (UDP-2,3-diacetamido-2,3-dideoxy-α-D-mannuronate biosynthesis) contains several enzymes wbpA, wbpB, wbpD, and wbpI that are shown to be significantly elevated in current smokers (K13015, K13019, and K13018). We also found that PWY6895, which is a part of thamin biosynthesis, is significantly changed in smokers. When taken together, the changed riboflavin metabolism revealed by metabolomic analysis, we speculated that a change in vitamin B metabolism may be associated with active smoking. Besides, the phenotype analysis revealed that some key enzymes involved in nicotinate and nicotinamide metabolism (KEGG database: ko00760) were related to smoking status, including elevated nicotinamide-nucleotide adenylyltransferase (K00952), enamidase (K15358), and 2-hydroxymethylglutarate dehydrogenase (K19647). Some sphingolipids-related enzymes were also elevated in smokers, including homoserine phosphotransferase (K02203) and histo-blood group ABO system transferase (K00709). The relationship between these two key enzymes and the sphingolipids metabolism is shown in Figure 4A. Correlations between the OTUs and predicted pathways as well as important enzymes are shown in Figure 4B. By offering insights into the possible function of microbial community and the relationship with metabolome, we believe that gut microbiota has contributed to the changed metabolomic profile in smokers.

### Multi-Omics Analysis Reveals the Relationship Between the Gut Microbiota and Serum Metabolites Associated With Smoking Status

**Figure 5:**
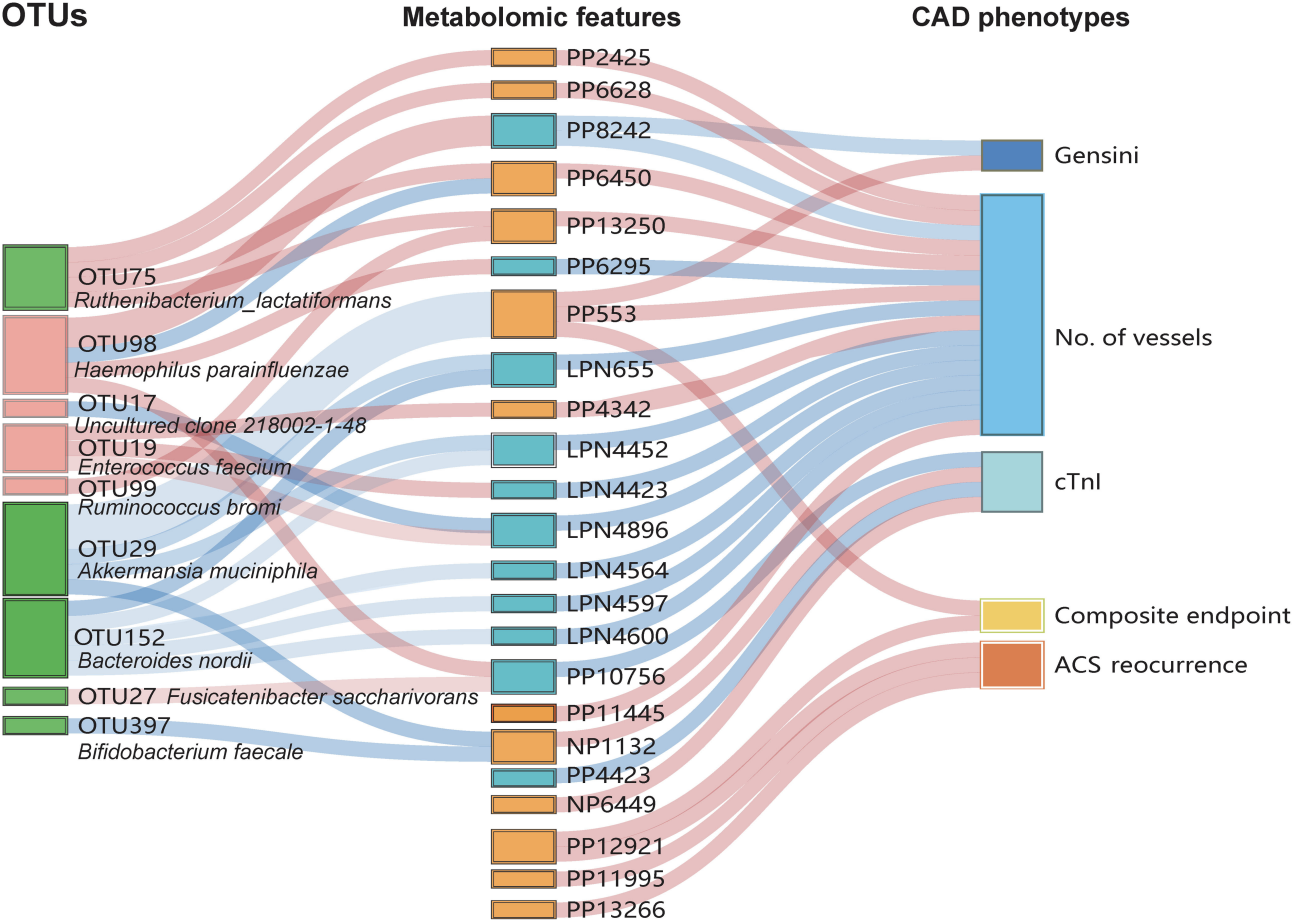
Interrelationship among smoking-associated gut flora, serum metabolic features, and major CAD phenotypes. A Sankey plot was utilized to examine and visualize the correlation between important smoking-associated OTUs and the severity and outcomes of CAD in the mediation of key serum metabolic features. Connections in red indicate positive correlations while blue connections indicate negative correlations (Spearman correlation analysis, *p* < 0.05). In the left column, red boxes indicate smoking-positive OTUs, and green boxes indicate smoking-negative OTUs. In the metabolic features column, orange boxes indicate smoking-positive metabolic features and blue boxes indicate smoking-negative metabolic features. A composite endpoint was defined as all-cause mortality and/or reoccurrence of ACS and/or readmission for cardiac causes. No. of vessels: ANthe number of stenosed vessels; cTnI: cardiac troponin I.

We subsequently assessed the correlation between the gut microbiota and serum metabolites to further explore the interrelationship between gut microbiota, metabolomic features, and clinical phenotypes associated with smoking. As demonstrated in Figure 5, a total of nine smoking-associated OTUs that contributed were significantly (*p* < 0.05) correlated with 18 metabolomic features, which were further correlated with indicators of disease severity and/or the clinical outcome. At the OTU level, we observed that some of the smoking-positive OTUs were found to be positively correlated with poor CAD phenotypes possibly through the mediation of metabolites. Notably, OTU 99 and OTU 19 were positively correlated to disease severity by mediating PP13250 and PP4342, respectively. Moreover, we also discovered some negative relationships between some of the smoking-positive metabolites and smoking-negative OTUs (PP553 with OTU 29; NP1132 with OTU 29 and OTU 397), implying that accumulation of pernicious metabolites may be related to the depletion of potentially beneficial bacteria. Whereas, we were not able to find the correlation between some of the important metabolites with any of the discriminant OTUs, such as PP12921 that is tightly correlated to poor clinical prognosis. This implies that cigarette smoking may exert an influence on metabolomic and microbial features of individuals through more diversified and complicated mechanisms. The results of the multi-omics analysis are summarized in Supplementary Table S7.

Overall, the smoking-associated microbial and metabolic features are shown above may provide further evidence of the microbial dysbiosis and changed metabolomic profile in CAD smokers, which has the potential to explain the cross talk of gut-heart axis in the pathogenesis of CAD.

## Discussions

In response to environmental perturbations such as cigarette smoking, bacteria in the human gut may thrive or decline as a functional community. We demonstrated that smoking patients with CAD had significantly different gut microbiota composition and serum metabolomic profiles compared with never smokers. Besides, through multi-omics correlative study, our study found that these discriminant microbe features and metabolomic features were correlated and also correlated well with clinical indicators.

We evaluated the smoking-related microbial change sequentially from phylum to OTU level. From a relatively macro perspective, we discovered an increasing gradient of the ratio of Firmicutes phylum in current smokers compared with former and never smokers. As the most abundant phylum, it was reported that the ratio of Firmicutes is increased in active smokers but can shift back after smoking cessation (16). The ratio of Firmicutes to Bacteroidetes in patients with CAD is higher than in healthy controls (32) and in the present study, a negative correlation was found between *Bacteroidetes* and inflammation indicators. Furthermore, the LEfSe analysis was applied to identify differential bacteria composition between smoking and non-smoking patients with CAD. We found out some smoking-negative genera (*Lactobacillus* and *Veillonella)* as well as some smoking-positive taxa (*Desulfovibrionaceae* and *Eubacterium)*. A similar pattern of increased *Desulfovibrionaceae* and decreased *Lactobacillus* spp. were previously observed when mice were fed a high-fat “Western” diet (33). *Desulfovibrionaceae* may have pro-inflammatory effects for their sulfate-reducing capacities producing toxic hydrogen sulfide (H_2_S). Ijssennagger presented that H_2_S produced by gut bacteria may damage the intestinal mucus layer by reducing disulfide bonds, resulting in the invasion of toxins and stimulation of host inflammation (34). *Lactobacillus* and *Eubacteria* are both BSH-producing genera, which may act as cholesterol-lowering agents by deconjugating bile salts and decrease cholesterol reabsorption (35). Besides, specific *Eubacteria* spp. has bacterial 7alpha-dehydroxylases to convert primary BAs to secondary BAs [deoxycholic acid (DCA) and lithocholic acid (LCA)] (36). Secondary bile acids can be reabsorbed and impact host lipid and glucose metabolism through several nuclear receptors [liver X receptor (LXR), pregnane X receptor (PXR), and specific G-protein-coupled receptors (GPCRs) like takeda G-protein-coupled receptor 5 (TGR5)] (37–39). Quite surprisingly, the depletion of several bile acids and taurine were significant in the current smokers, implying a changed bile acids profile associated with cigarette smoking. The previous study has shown that smoking might break the homeostasis of bile acids metabolism (40). Based on the above analysis, we believe that further investigations are acquired to explore the causal link between smoking, gut microbiota, and bile acids metabolism.

At the OTU level, a previous study conducted in a healthy population suggested that the smoking-related microbiota composition profile features the increased *R. bromii* and depleted *A. muciniphila* and *B. nordii*, which is in line with our study in CAD smokers (15). We found via intergroup comparison that the OTUs enriched in current smokers comprise several opportunistic pathogens such as *H. parainfluenzae* and *Klebsiella sp*. The gram-negative bacteria infection can induce cytokine burst by releasing lipopolysaccharide (LPS) to affect the plaque stability and also the development of atherosclerosis (41). *Klebsiella* was reported to be associated with the hypertensive population and may play a part in hypertensive progression (42). The major discriminant feature of the current smokers may be the deprivation of some potentially beneficial taxa, such as *A. muciniphila* and *Bifidobacterium* spp. *A. muciniphila* is considered to have multiple probiotic roles in host metabolic modulation, immune regulation, and gut barrier protection (43). The protective role of *A. muciniphila* against atherosclerosis is also promising due to its lipid-lowering and anti-inflammation abilities (44, 45). *B. catenulatum* was reported to have an anti-inflammation role and to assist other probiotics to produce butyrate (46, 47). Other possibly beneficial bacteria depleted in the smoker population include *Fusicatenibacter saccharivorans and Collinsella aerofaciens. F. saccharivorans* was reported to have an anti-inflammation role to relieve ulcerative colitis (UC) in the murine model, while a novel subsp. of *C. aerofaciens* was isolated and proved to be capable of butyrate synthesis (48, 49). Some probiotic strains have been investigated to exhibit beneficial effects on CAD (50). For instance, a 12-week intake of *Lactobacillus rhamnosus* GG (LGG) exhibited beneficial effects in reducing mega inflammation and metabolic endotoxemia in participants with CAD (51). Moreover, co-supplementation of probiotics (LGG) and prebiotic inulin in subjects with CAD for 8 weeks had beneficial effects on depression, anxiety, as well as inflammatory biomarkers (52). The underlying mechanisms of probiotics on CAD are complicated and are yet to be elucidated.

The human gut microbial ecosystem is now considered an endocrine organ, which interacts intensively with the host through circulating metabolites. Metabolomics analysis also revealed the significant change in patients with CAD with different smoking statuses. As presented above, several sphingolipids were found to be elevated in current smokers, including Cer(d18:1/22:0) (PLP28), Cer(d18:1/24:0) (PLP67), 2 glucosylceramides (PLP379 & PLP2763), and sphinganine (PP11445). In pathway analysis conducted by MetaboAnalyst 4.0, sphingolipids metabolism in current smokers was significantly changed compared with the never smokers; and the significant difference between former and never smokers revealed the partial irreversibility of this change. Prior to this study, Tong et al. (53) has elucidated that cigarette smoking can interfere with insulin secretion through induction of ceramide accumulation and activation of oxidative stress. Also, the detrimental effect continued even during smoking cessation, which is consistent with the irreversibility presented in our study. Animal experiments also confirmed the changed ratio of Cer(d18:1/24:0) to Cer(d18:1/18:0) as markers of CS exposure in the lungs, plasma, and liver (54). As the metabolites of sphingolipids, ceramides are considered as lipotoxic inducers of disturbed glucose homeostasis and also an active player in the progression of atherosclerosis (55). Cer(d18:1/22:0) and Cer(d18:1/24:0) are both associated with stroke severity at admission and future risk (56). Studies in rodent models revealed that the inhibition of ceramide synthesis reduces ischemic cardiomyopathy-related heart failure post-MI or tissue hypoxia and preventing ventricular remodeling (57). According to Edsfeldt et al. (58), six sphingolipids (particularly GluCer) can boost plaque inflammation and promote vascular smooth muscle cell apoptosis.

In recent decades, the gut-heart axis has emerged as a novel concept and provided new insights into atherosclerotic pathogenesis. Previous studies have shown that an imbalance in the gut-heart axis due to the gut microbiota plays an important role in atherosclerosis progression. The gut microbiota promotes the development of atherosclerosis by producing intermediate metabolites, including TMAO, LPS, Phenylacetylglutamine (PAGln), and reducing SCFAs (59). This theory may also help to explain the discovered gut microbiota change and elevated ceramide level that may be related to different CAD prognoses. A prior study of our group has convincingly shown that intestinal farnesoid X receptor (FXR) may modulate atherosclerosis by elevating ceramide metabolism (60). FXR was identified as an orphan nuclear receptor that plays multiple roles in regulating bile acid homeostasis, lipid, and glucose metabolism (61). Noticing the possible influence on the bile acid profile of smoking as mentioned above, we speculated that the ceramide and bile acid dysregulation may be related to FXR. The activation of intestinal FXR can decrease bile acid absorption, while hepatic FXR has a role in attenuating cholesterol metabolism/bile acid synthesis by suppression of CYP7A1 and CYP8B1 expression, both contributing to a decreased level of circulating bile acids (62). Intestinal FXR activation also induces genes involved in ceramide synthesis that potentiate metabolic disorders (63). Several therapeutic strategies have been designed to improve metabolic diseases by inhibiting FXR activity. For instance, metformin, tempol, or antibiotics can reduce the abundance of BSH-secreting gut microbiota, and thus increase levels of endogenous FXR antagonists [especially tauro-β-muricholic acid (T-β-MCA)] (64, 65). Also, direct oral administration of FXR antagonists including ursodeoxycholic acid and Gly-MCA can affect bile acid and lipid metabolism (66, 67). A previous publication has indicated a potential association between smoking and FXR in pulmonary inflammation (68). The interaction of cigarette smoking, gut microbiota composition shift, and ceramide and bile acids dysregulation needs to be further elucidated.

Despite *de novo* generation in mammalian tissue and dietary uptake, sphingolipids can also be produced by the *Bacteroidetes* spp., which is one of the dominant phyla of the gut microbiome (on an average constitute of 30–40%) (69). Bacteroidetes have the necessary enzyme serine palmitoyl-transferase (SPT), making them the only gut commensal group known to produce sphingolipids (70). Recent studies have shown that deficiency of Bacteroidetes-derived sphingolipids can affect host sphingolipid metabolism resulting in elevated ceramide levels and subsequent amplification of host inflammation (71, 72). In our study, several OTUs under the Bacteroidetes phylum were depleted (e.g., *Bacteroides nordii* and *Prevotella copri*) in smokers compared with never smokers. Although no significant change was detected in the abundance of Bacteroidetes, the negative correlation between Bacteroidetes and sphinganine (PP11445, positively correlated with the number of stenosed vessels) may suggest the possible interrelation. Cigarette smoking may play a role in promoting vulnerable plaque formation through interfering with gut microbiota sphingolipid production and regulating host sphingolipid levels. Possible therapeutic targets on *Bacteroidetes*-derived sphingolipids may be beneficial for both current and former smokers.

We also noticed a decreased level of L-glutamate and L-glutamine in current smokers, while the difference is no more significant between former and never smokers. Besides, pathway analysis revealed a changed D-glutamine and D-glutamate metabolism, which is in line with the microbial functional prediction conducted by PICRUSt2. In the nervous system, glutamate is an important excitatory transmitter and plays an important role in the addiction to nicotine and other drugs. Cigarette smoking was found to be associated with decreased regional brain glutamate as well as circulating glutamate (73, 74). However, third-hand smoking and alcohol consumption can also induce imbalanced Glu-Gln metabolism, making the issue more complicated (75). When ACS occurs, glutamate is important in energy metabolism and promoting survival of cardiac cells subjected to hypoxia/reoxygenation (76). Glutamine was reported to inhibit the progression of atherosclerosis and promote plaque stability by activating M2 macrophages (77). The downregulated Gln and Glu levels in current smokers may need more investigation to possibly improve the post-ACS prognosis in smokers.

Results from epidemiological studies have identified cigarette smoking as a major risk factor for poor CAD prognosis. By the same time, evidence has shown that gut microbiota might play an important role in CADs. However, to our knowledge, this is the first multi-omics study to investigate the role of smoking in atherosclerosis pathogenesis. Our results showed that certain alterations in the gut microbial community and serum metabolites are related to smoking status. Compared with simple mono-omic microbiome analysis, the addition of metabolome study can directly reflect the functional capacity of symbiont gut flora by circulating microbe-related metabolites. Based on these differential bacteria and metabolites, we are provided with novel biomarkers or therapeutic targets for CAD progression beyond the traditional concepts like smoking-induced endothelial dysfunction. Taken the heavy coronary disease burden and high global smoking prevalence into consideration, our study may open up the possibility of modulating gut microbiota to improve CAD prognosis in smokers and even high-risk former or never smokers. To further explore the interaction of smoking and smoking-related microbes, functional studies are urgently needed.

Our study has some limitations as well. First, due to the small proportion of current and former smokers in female patients with CAD, we excluded all the female participants to avoid possible confounding factors in our study. An intriguing epidemiological issue is the vulnerability of female smokers to develop CADs. The pooled adjusted female-to-male relative risk ratio of smoking compared with non-smoking for CAD was 1.25; however, the possible mechanism remains unclear (78). It was also reported that the influence of smoking status on the metabolomic profile may be gender-specific (79). To make sure how smoking status interacts with cardiovascular disease progression, the female population should be taken into consideration. Another limitation of our study is the unmeasurable influence of passive smoking. Passive smoking can also have a detrimental effect on cardiovascular health but it is hard to be measured or documented (80).

## Conclusions

Our study demonstrated that smoking can influence the physical condition of patients with CAD from a multi-omics perspective. Gut microbiota analysis revealed that smoking may influence the composition of host gut flora by increasing potentially pathogenic bacteria such as *Desulfovibrionaceae, H. parainfluenzae*, and *Klebsiella*, and reducing possibly beneficial bacteria such as *Bifidobacterium* spp. and *A. muciniphila*, thus increasing the metabolic risk of CAD smokers. The metabolomic study showed that smoking is associated with concentration variations in sphingolipids, glycerophospholipids, and amino acid metabolism. Moreover, the serum metabolite profile of smokers is partially reversible after stopping smoking, which indicates the benefits of smoking cessation to improve CAD prognosis. Our findings provide new insights into the heterogenic roles of cigarette smoking and the multi-omics interactions in CAD, and the identified microbiota or metabolites may serve as biomarkers of smoking cessation status or novel therapeutic targets. However, more functional and interventional studies will be needed to elucidate the role of smoking in CAD pathogenesis and progression.

## Data Availability Statement

The datasets presented in this study can be found in online repositories. The names of the repository/repositories and accession number(s) can be found below: https://www.ncbi.nlm.nih.gov/, SRP167862.

## Ethics Statement

The studies involving human participants were reviewed and approved by the Ethics Review Board of the Peking Union Medical College Hospital, Chinese Academy of Medical Sciences. The patients/participants provided their written informed consent to participate in this study.

## Author Contributions

XH and SZ designed and supervised the study. XH and YF managed the clinical research. XH, YF, HL, RZ, XZ, and YS obtained the samples and clinical information. YF and XH performed the data analysis. YF, XH, and SZ wrote, reviewed, and revised the manuscript. All the authors read and approved the final manuscript.

## Funding

This study was supported by the Beijing Natural Science Foundation (Grant No. 7202152), the National Natural Science Foundation of China (Grant Nos. 81670329 and 81974183), the CAMS Innovation Fund for Medical Sciences (CIFMS) (Grant Nos. 2017-I2M-2-002, 2016-I2M-1-002, and 2019-I2M-1-001), the National Key Research and Development Program of China (Grant No. 2016YFC0901502), and Center for Rare Diseases Research, Chinese Academy of Medical Sciences, Beijing, China (Grant No. 2016ZX310174-4).

## Conflict of Interest

The authors declare that the research was conducted in the absence of any commercial or financial relationships that could be construed as a potential conflict of interest. The reviewers H-ZC and JS declared a shared affiliation with the authors to the handling editor at the time of the review.

## Publisher's Note

All claims expressed in this article are solely those of the authors and do not necessarily represent those of their affiliated organizations, or those of the publisher, the editors and the reviewers. Any product that may be evaluated in this article, or claim that may be made by its manufacturer, is not guaranteed or endorsed by the publisher.
