# Supplementary figures and images for: Impacts of Cigarette Smoking Status on Metabolomic and Gut Microbiota Profile in Male Patients With Coronary Artery Disease: A Multi-Omics Study
Source: Front Cardiovasc Med. 2021 Oct 28;8:766739. doi: 10.3389/fcvm.2021.766739 (PMC8581230; doi:10.3389/fcvm.2021.766739)

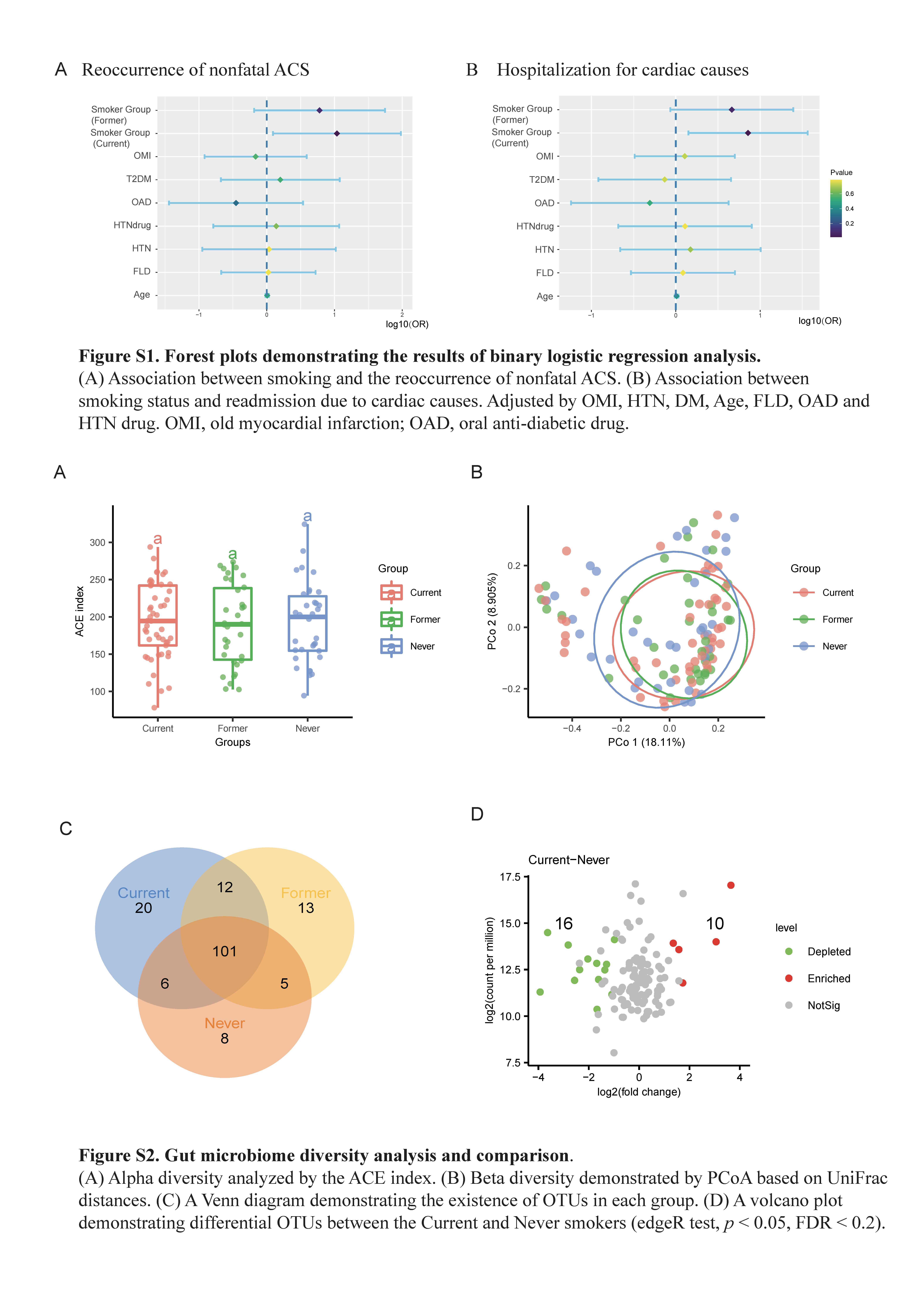

Supplement: Supplementary file 1 [file Image_1.TIFF]

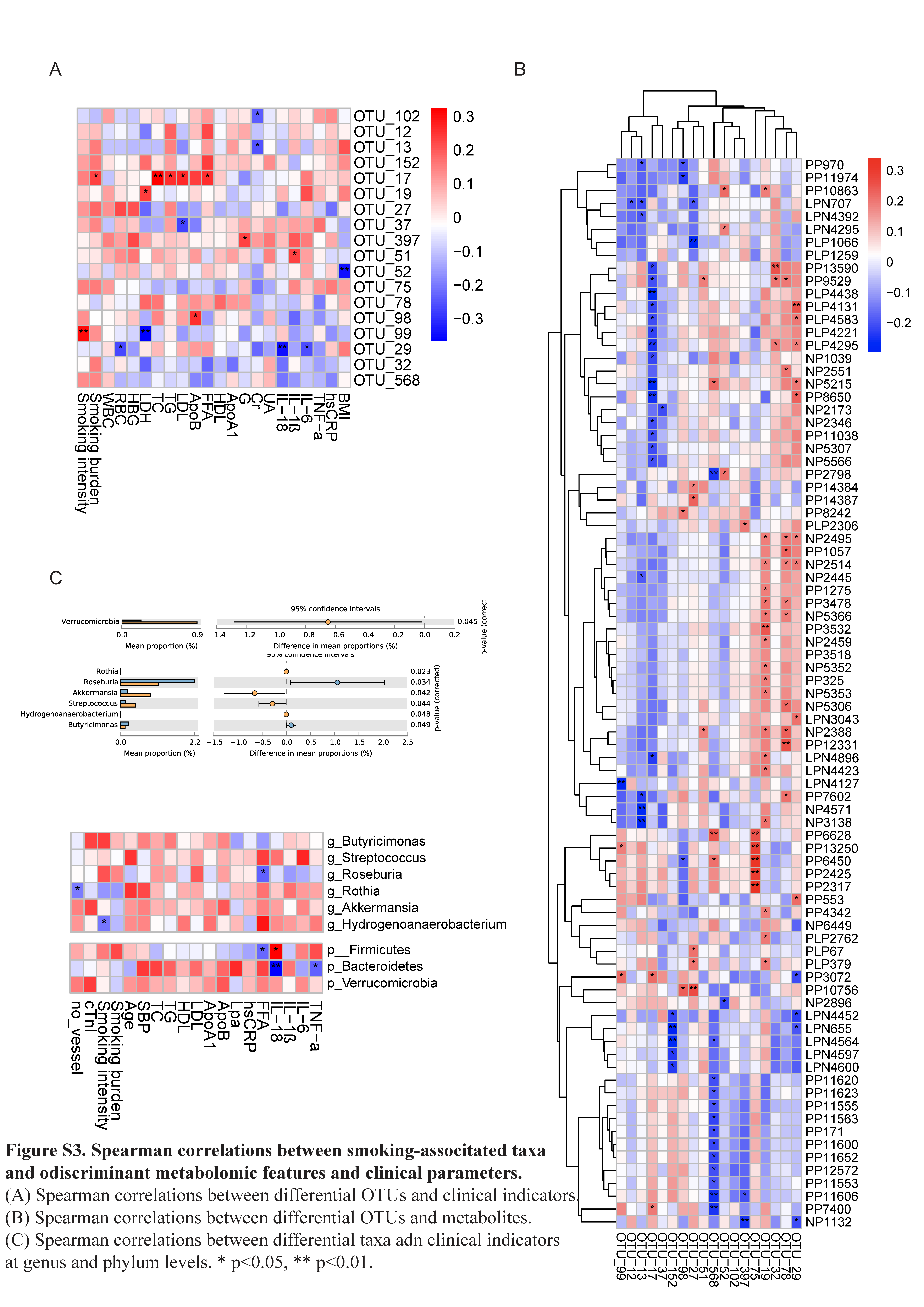

Supplement: Supplementary file 2 [file Image_2.TIFF]

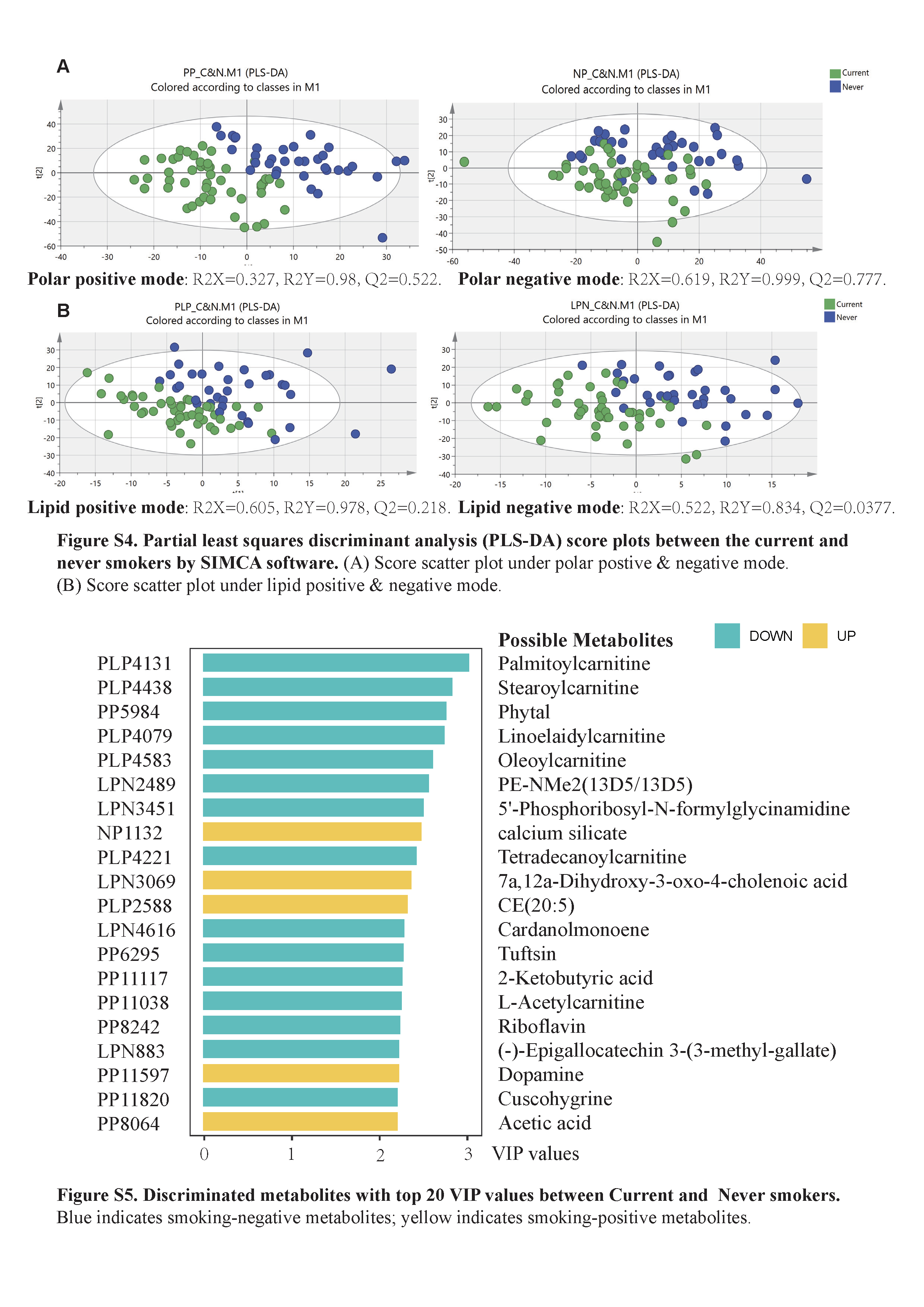

Supplement: Supplementary file 3 [file Image_3.TIFF]

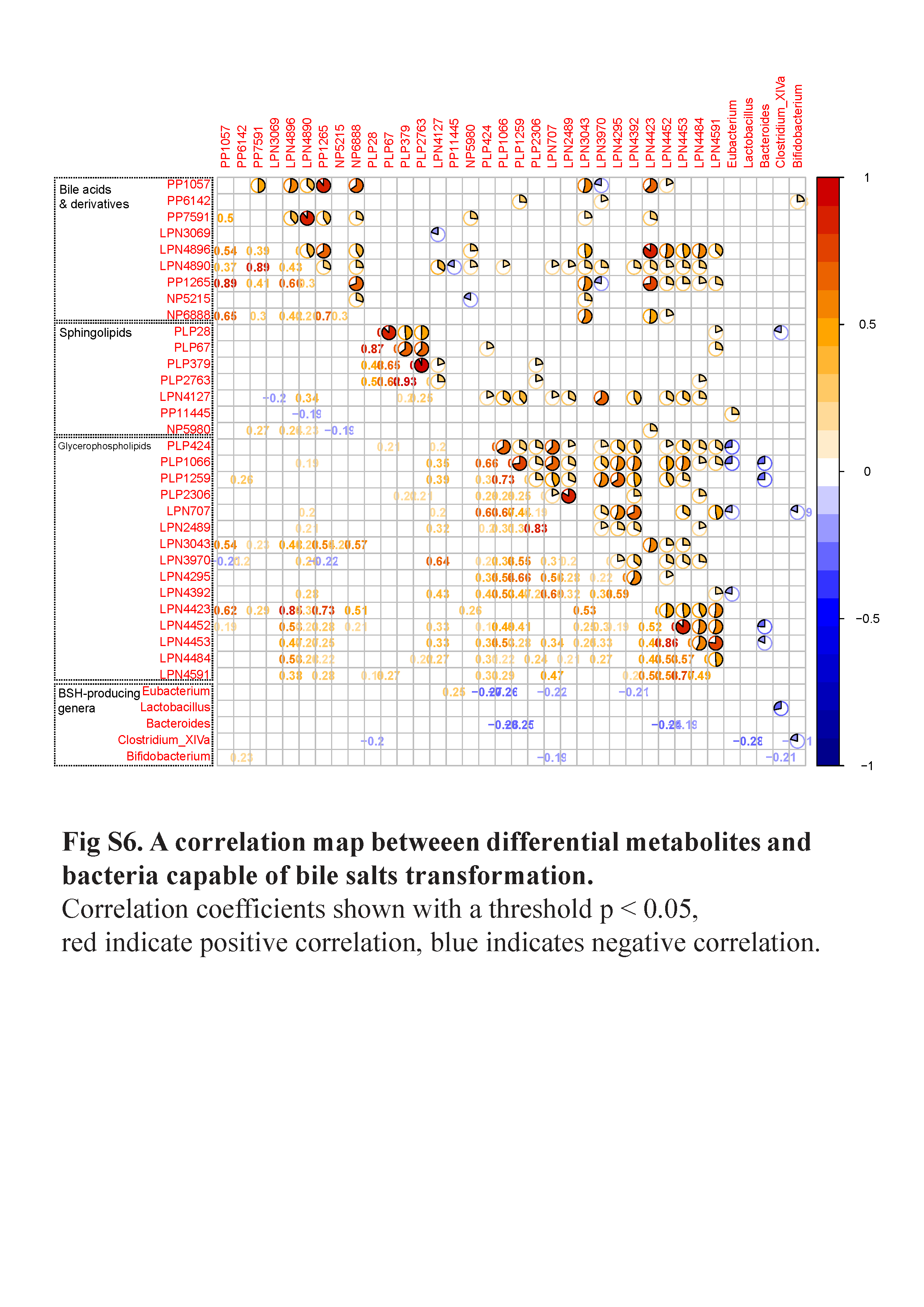

Supplement: Supplementary file 4 [file Image_4.TIFF]

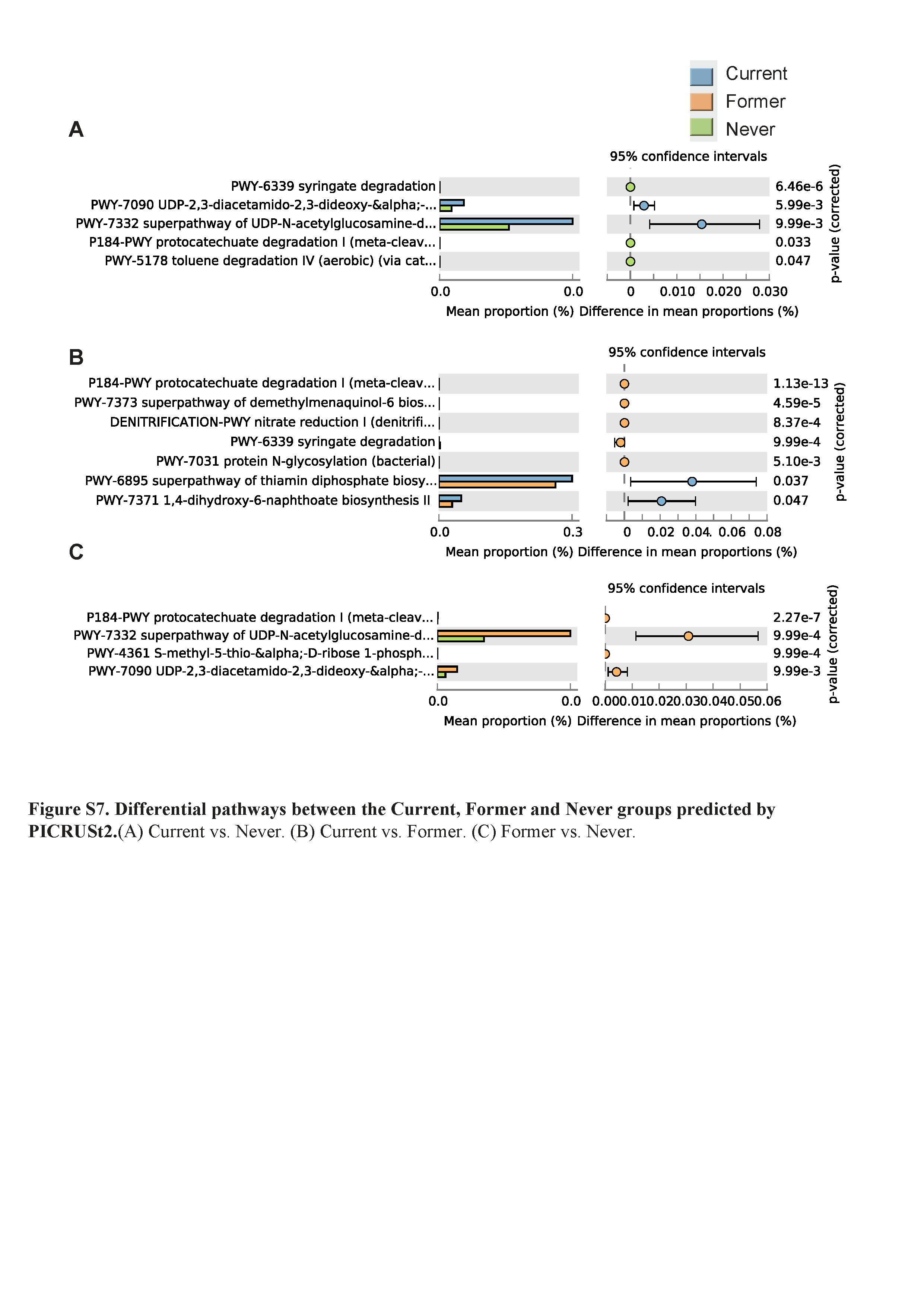

Supplement: Supplementary file 5 [file Image_5.TIFF]

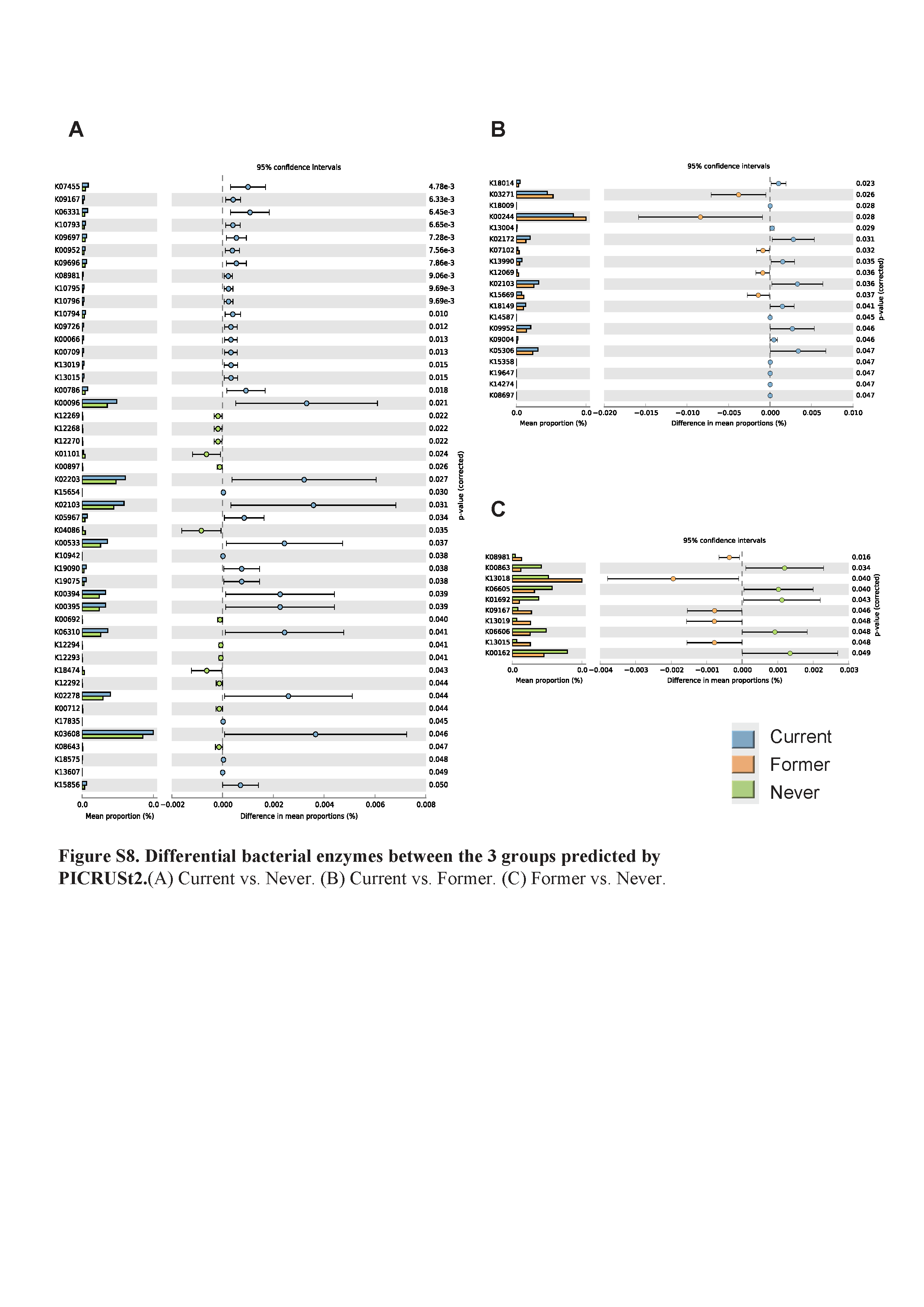

Supplement: Supplementary file 6 [file Image_6.TIFF]
